# SK channel activation is neuroprotective in conditions of enhanced ER–mitochondrial coupling

**DOI:** 10.1038/s41419-018-0590-1

**Published:** 2018-05-22

**Authors:** Birgit Honrath, Inge E. Krabbendam, Carmen IJsebaart, Valentina Pegoretti, Nadia Bendridi, Jennifer Rieusset, Martina Schmidt, Carsten Culmsee, Amalia M. Dolga

**Affiliations:** 1 Institute of Pharmacology and Clinical Pharmacy, University of Marburg, 35043 Marburg, Germany; 2 Faculty of Science and Engineering, Groningen Research Institute of Pharmacy (GRIP), Research School of Behavioural and Cognitive Neurosciences (BCN), Department of Molecular Pharmacology, University of Groningen, 9713 AV Groningen, The Netherlands; 3 INSERM U1060, INRA U1235, Laboratoire CarMeN, Lyon University, Université Claude Bernard Lyon1, INSA-Lyon, F-69921 Oullins, France

## Abstract

Alterations in the strength and interface area of contact sites between the endoplasmic reticulum (ER) and mitochondria contribute to calcium (Ca^2+^) dysregulation and neuronal cell death, and have been implicated in the pathology of several neurodegenerative diseases. Weakening this physical linkage may reduce Ca^2+^ uptake into mitochondria, while fortifying these organelle contact sites may promote mitochondrial Ca^2+^ overload and cell death. Small conductance Ca^2+^-activated K^+^ (SK) channels regulate mitochondrial respiration, and their activation attenuates mitochondrial damage in paradigms of oxidative stress. In the present study, we enhanced ER–mitochondrial coupling and investigated the impact of SK channels on survival of neuronal HT22 cells in conditions of oxidative stress. Using genetically encoded linkers, we show that mitochondrial respiration and the vulnerability of neuronal cells to oxidative stress was inversely linked to the strength of ER–mitochondrial contact points and the increase in mitochondrial Ca^2+^ uptake. Pharmacological activation of SK channels provided protection against glutamate-induced cell death and also in conditions of increased ER–mitochondrial coupling. Together, this study revealed that SK channel activation provided persistent neuroprotection in the paradigm of glutamate-induced oxytosis even in conditions where an increase in ER–mitochondrial coupling potentiated mitochondrial Ca^2+^ influx and impaired mitochondrial bioenergetics.

## Introduction

Multiple lines of evidence indicate that the etiologies of neurodegenerative disorders, such as Alzheimer’s disease (AD) or Parkinson’s disease (PD) are strongly associated with common features of neuronal damage such as dysregulation of calcium (Ca^2+^) homeostasis and oxidative stress^1–5^. Disrupted Ca^2+^ homeostasis can lead to mitochondrial Ca^2+^ ([Ca^2+^]_m_) overload, and subsequently to an impairment of mitochondrial energy metabolism and respiration^6,7^. Under physiological conditions however, [Ca^2+^]_m_ is a prerequisite for aerobic energy metabolism through the regulation of oxidative phosphorylation (OXPHOS) and mitochondrial ATP production^8,9^.

Close spatial interactions between the endoplasmic reticulum (ER) and mitochondria are essential for rapid and sustained [Ca^2+^]_m_ uptake. These close contacts are established at the so called mitochondria-associated ER membranes (MAM), thereby facilitating Ca^2+^ transfer between ER and mitochondria through mitochondrial voltage-dependent anion channels (VDAC) and ER-located inositol-1,4,5-trisphosphate receptors (IP_3_R), which are physically connected by glucose-regulated protein 75 (GRP75)^10–12^. Mutations in MAM-associated proteins have been identified to either enhance or reduce ER–mitochondrial coupling (EMC), thereby leading to dysregulation of MAM interfaces and progressive neuronal degeneration as shown in models of AD and amyotrophic lateral sclerosis^13–15^.

In neuronal cells, activation of small conductance Ca^2+^-activated K^+^ (SK) channels regulated Ca^2+^ uptake and retention in the ER^16^, and also controlled mitochondrial Ca^2+^ homeostasis and respiration^17^. Activation of SK channels in conditions of ER stress and glutamate-induced oxidative stress (oxytosis) preserved cell viability, and restored ER and mitochondrial function, respectively. In this study, we aimed to investigate the ability of SK channels to confer protection following oxidative stress in conditions where EMC was increased.

## Results

### Inducing a physical linkage between ER and mitochondria

ER and mitochondria temporarily and dynamically form close contacts at the MAM, thereby allowing for the exchange of proteins, lipids, and ions between the organelles^18^. To study the structure and function of ER–mitochondrial connections, we used genetically encoded bifunctional linkers^19^ that both, tighten the contact between ER and mitochondria, and expand the MAM interface area. These linkers consist of OMM-FKBP12-mRFP and ER-targeted-FRB-CFP fusion proteins which heterodimerize in response to rapamycin application at very low concentrations (100 nM).

**Fig. 1:**
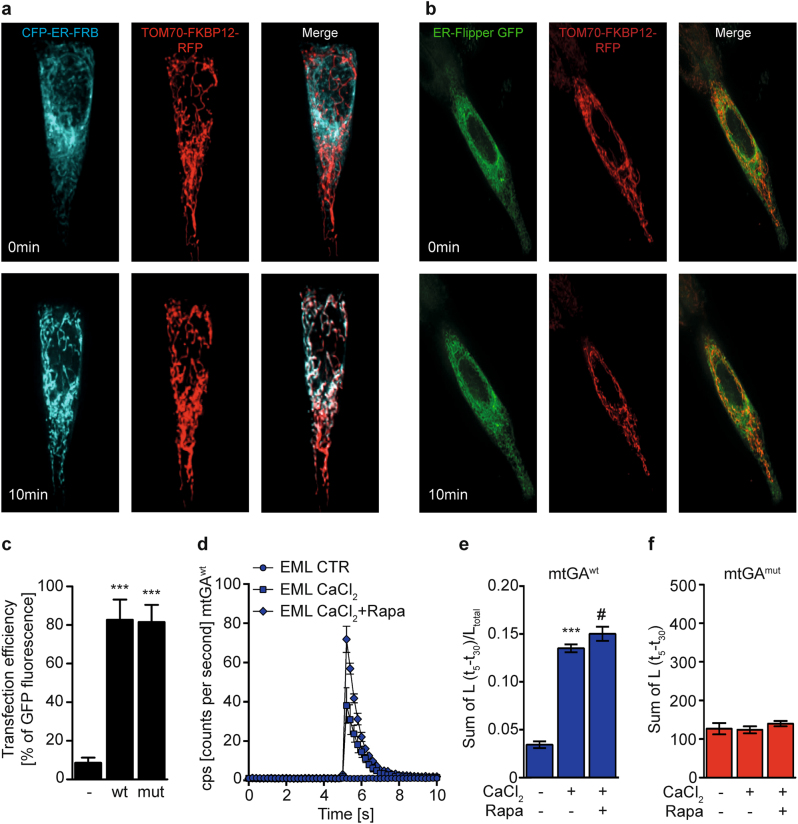
Inducing ER–mitochondrial associations in neuronal HT22 cells increases [Ca^2+^]_m_ uptake. **a**, **b** Representative fluorescent images of an individual HT22 cell expressing (**a**) ER–mitochondrial linkers or (**b**) ER-Flipper-GFP and TOM70-FKBP12-RFP prior to and 10 min following rapamycin (100 nM) addition. Fluorescent traces are shown individually (left and middle panels) and as an overlay of both fluorescent channels (right panels). Cyan: ER-CFP-9xFRB, green: ER-Flipper-GFP, red: TOM70-FKBP12-RFP. **c** Transfection efficiency of HEK293T cells transfected with mtGA^wt^ or mtGA^mut^. Data are presented as mean ± SD, *n* = 8–9, Student’s *t*-test, ***p < 0.0001. **d** Representative measurement of [Ca^2+^]_m_ uptake in HEK293T cells transfected with mtGA^wt^ stimulated with buffer (CTR) or 50 mM CaCl_2_ in the presence or absence of 100 nM rapamycin. Data are presented as mean ± SD, *n* = 3–4. **e**, **f** Quantification of [Ca^2+^]_m_ in HEK293T cells expressing (**e**) mtGA^wt^ or (**f**) mtGA^mut^ following stimulation with buffer or 50 mM CaCl_2_ with/without 100 nM rapamycin. Luminescence in mitoGA^wt^ stimulated cells was normalized to the total luminescence (*L*_*t* = 5–30_/*L*_total_). Data are presented as mean ± SD, Student’s *t*-test*, n* = 3-4, ***p < 0.0001 compared to untreated control, ^#^*p* < 0.05 compared to 50 mM CaCl_2_ without rapamycin

Upon application of rapamycin, we observed a time-dependent co-localization of CFP-tagged ER with RFP-tagged mitochondria indicating the induction of EMC, reaching complete co-localization after 10 min following rapamycin treatment (Fig. 1a). In contrast, a GFP-tagged ER-Flipper control plasmid (FL), co-transfected with OMM-FKBP12-mRFP, failed to co-localize with RFP-tagged mitochondria following rapamycin treatment (Fig. 1b), which confirmed the specificity of the hereafter called ER–mitochondrial linkers (EML).

### Enhancing EMC increases [Ca^2+^]_m_ uptake and impairs the mitochondrial reserve respiratory capacity

The rapamycin-induced heterodimerization of the genetically encoded linkers tightens ER–mitochondrial contact points and increases the surface of the MAM interface^19^, which might amplify Ca^2+^ transfer into the mitochondrial matrix and modulate mitochondrial respiration^20^.

To this end, we assessed [Ca^2+^]_m_ uptake in the presence of the linkers and their pharmacological inducer rapamycin. In order to study fast changes in [Ca^2+^]_m_ uptake induced by strengthening EMC, we utilized a system to perform real-time [Ca^2+^]_m_ uptake measurements in HEK293T cells transfected with the linkers and a mitochondrial GFP-aequorin^21^. Up to 80% of HEK293T cells on average were transfected with the wild type (mtGA^wt^) or a Ca^2+^-binding deficient mutant aequorin version (mtGA^mut^) serving as an internal control (Fig. 1c). Calibration of the aequorin sensor revealed a dose-dependent increase in the luminescence with increasing CaCl_2_ concentrations (Figure S1a-b). Stimulation of HEK293T cells transfected with the linkers and co-expressing mtGA^wt^ with 50 mM CaCl_2_ induced [Ca^2+^]_m_ uptake as shown by an elevation of the luminescence signal (Fig. 1d) and of the total luminescence (Fig. 1e) compared to cells stimulated with control solution. Importantly, stimulation of linker-transfected HEK293T cells which were pre-treated with 100 nM rapamycin during sensor reconstitution, resulted in higher [Ca^2+^]_m_ uptake compared to control and untreated cells, suggesting that EMC strengthening increased [Ca^2+^]_m_ uptake. In contrast, [Ca^2+^]_m_ uptake in cells transfected with the Ca^2+^-binding deficient mutant (mtGA^mut^) was unchanged, indicating the specific binding of Ca^2+^ to the mtGA^wt^ construct (Fig. 1f). The treatment of transfected HEK293T cells with rapamycin did not affect cell viability as measured by the MTT Assay (Figure S1c).

**Fig. 2:**
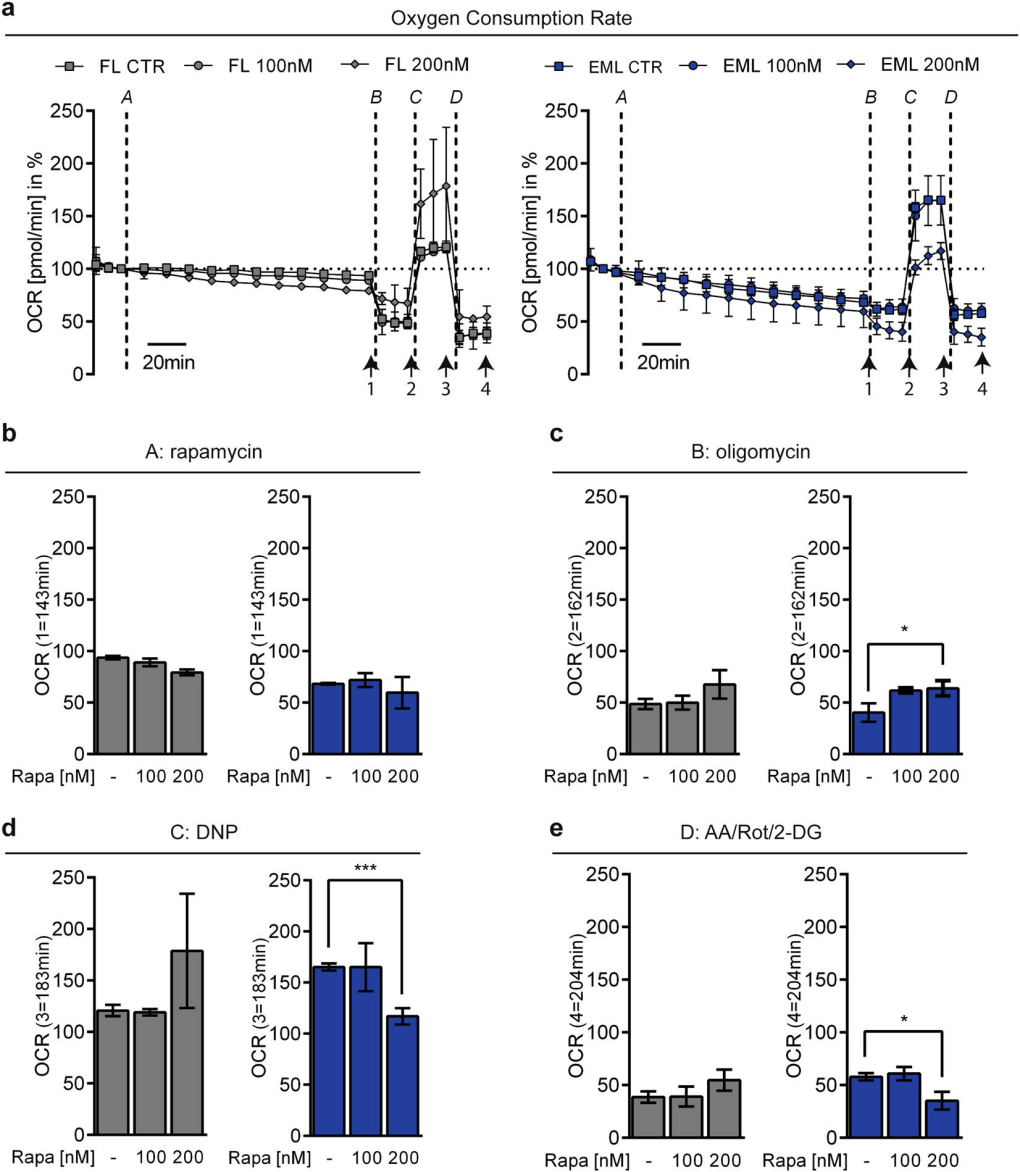
Enhancing ER–mitochondrial coupling impairs mitochondrial respiration. **a** Representative measurement of the oxygen consumption rate (OCR) in HT22 cells transfected with TOM70-FKBP12-mRFP and Flipper control plasmid (named FL, gray symbols/bars) or together with CFP-FRB-ER (named EML, blue symbols/bars) following the application of 100 nM rapamycin. A: rapamycin, B: oligomycin, C: DNP, D: antimycin A, rotenone, 2DG. Data are presented as mean ± SD, *n* = 3–6 per condition. Arrows indicate analyzed time points. **b**–**e** Quantification of the OCR at the indicated time points following the application of **b** rapamycin, **c** oligomycin, **d** DNP, and **e** antimycin A, rotenone, 2-DG. Student’s *t*-test, ***p < 0.0001 compared to untreated control

Next, we determined the effects of strengthening EMC on mitochondrial bioenergetics in HT22 cells using Seahorse extracellular flux analyses. Mitochondrial respiration, assessed as the oxygen consumption rate (OCR), was recorded in real-time following the application of 200 nM rapamycin over a period of 2 h. In cells transfected with the Flipper control construct (Fig. 2, gray symbols/bars), rapamycin application did change the OCR compared to untreated control cells. In contrast, rapamycin application to linker-transfected (Fig. 2, blue symbols/bars) cells did not change basal respiration (Fig. 2b) but seemed to affect ATP production (Fig. 2c), as assessed following oligomycin treatment. Interestingly, upon uncoupling of the respiratory chain using dinitrophenol (DNP), the induced maximal OCR was decreased only in linker-transfected cells in the presence of rapamycin compared to untreated control cells (Fig. 2d) indicating an effect on the reserve respiratory capacity. Final inhibition of mitochondrial complexes I (rotenone) and III (antimycin A), therefore, resulted in a lower OCR in linker-transfected cells where rapamycin was applied (Fig. 2e). Notably, the rapamycin-induced impairment of mitochondrial respiration in cells where EMC was increased, did not affect cell survival since cell proliferation was not impaired compared to Flipper-transfected control cells, as assessed using real-time impedance measurements (Figure S2a). Further, formation of the linker dimer did not affect ER integrity or function since the mRNA of *CHOP*, a classical marker for ER stress, was not changed following rapamycin treatment in either Flipper-transfected or linker-transfected cells (Figure S2b).

Taken together, we show that promoting EMC specifically amplified [Ca^2+^]_m_ influx, affected ATP production, and impaired the reserve respiratory capacity, without affecting basal respiration, cell viability, or proliferation.

### Strengthening EMC accelerates neuronal cell death

To study the consequences of increased EMC on neuronal cell death signaling, we induced oxidative stress and mitochondrial damage in neuronal HT22 cells by glutamate^22^.

**Fig. 3:**
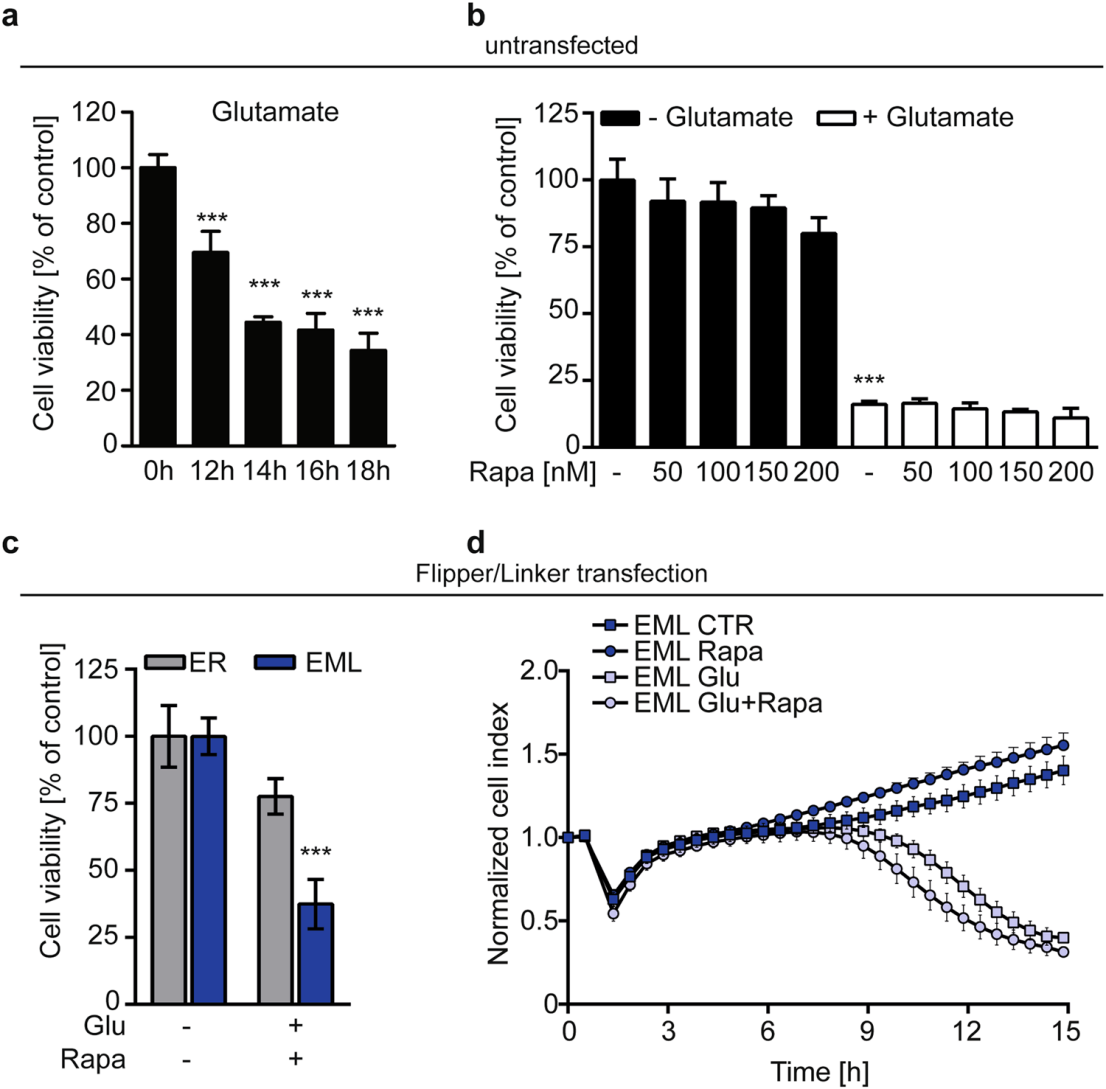
Strengthening EMC potentiates neuronal cell death. **a** MTT Assay following glutamate treatment (8 mM) for 12–18 h. Data are presented as mean ± SD, *n* = 8, Student's *t*-test ***p < 0.0001 compared to untreated control. **b** MTT assay following treatment with glutamate (8 mM, 16 h) in the presence or absence of rapamycin (0, 50, 100, 150, 200 nM). Data are presented as mean ± SD, *n* = 6, Student’s *t*-test ***p < 0.0001 compared to untreated control. **c** xCELLigence measurement of HT22 cells transfected with ER-FRB-CFP and TOM70-FKBP12-RFP (EML) following addition of glutamate (8 mM, 16 h) in the presence or absence of rapamycin (100 nM). Data are presented as mean ± SD, *n* = 6. **d** MTT Assay in HT22 cells transfected with TOM70-FKBP12-RFP and either ER-CFP (ER, gray bars) or ER-FRB-CFP (EML, blue bars) following treatment with glutamate (4 mM, 16 h) and rapamycin (100 nM). Data are presented as mean ± SD, *n* = 6, Student’s *t*-test ***p < 0.0001 compared to glutamate in ER control

In HT22 cells, cell death was detectable approximately 12 h following glutamate exposure with maximal damage detected after 16–18 h, as indicated by the MTT assay (Fig. 3a). Since rapamycin can induce mammalian target of rapamycin (mTOR) signaling and thereby autophagy^23^, we controlled for cell viability and cell death induction in the presence of rapamycin in neuronal HT22 cells. Rapamycin alone neither affected cell viability under control conditions nor glutamate toxicity (Fig. 3b), indicating that rapamycin at the low concentrations used in our study did not induce mTOR-dependent signaling nor autophagy.

Next, we investigated the consequences of rapamycin-mediated EMC, via linker heterodimerization, on cell viability using the MTT assay. Glutamate reduced cell viability in both, ER-transfected control and linker-transfected cells, yet cell death was pronounced in linker-transfected cells following rapamycin treatment (Fig. 3c, blue bars). We confirmed these pronounced effects on proliferation using real-time cellular impedance measurements showing that the addition of rapamycin to linker-transfected cells accelerated cell death induction by glutamate, represented as a drop in the cellular impedance curve (Fig. 3d). These results suggest that enhanced EMC accelerated glutamate-induced neuronal cell death.

### Activation of small conductance Ca^2+^-activated K^+^ (SK) channels preserves cell survival in conditions of enhanced EMC

Small conductance Ca^2+^-activated K^+^ (SK) channels have been identified in the ER membrane^16^, as well as in the inner mitochondrial membrane (IMM)^17^ of neuronal cells where they contribute to cell survival in conditions of stress. Their activation with the SK2/3 channel activator CyPPA^24^ reduced mitochondrial respiration, which prevented glutamate-induced mitochondrial dysfunction and cell death^17^.

**Fig. 4:**
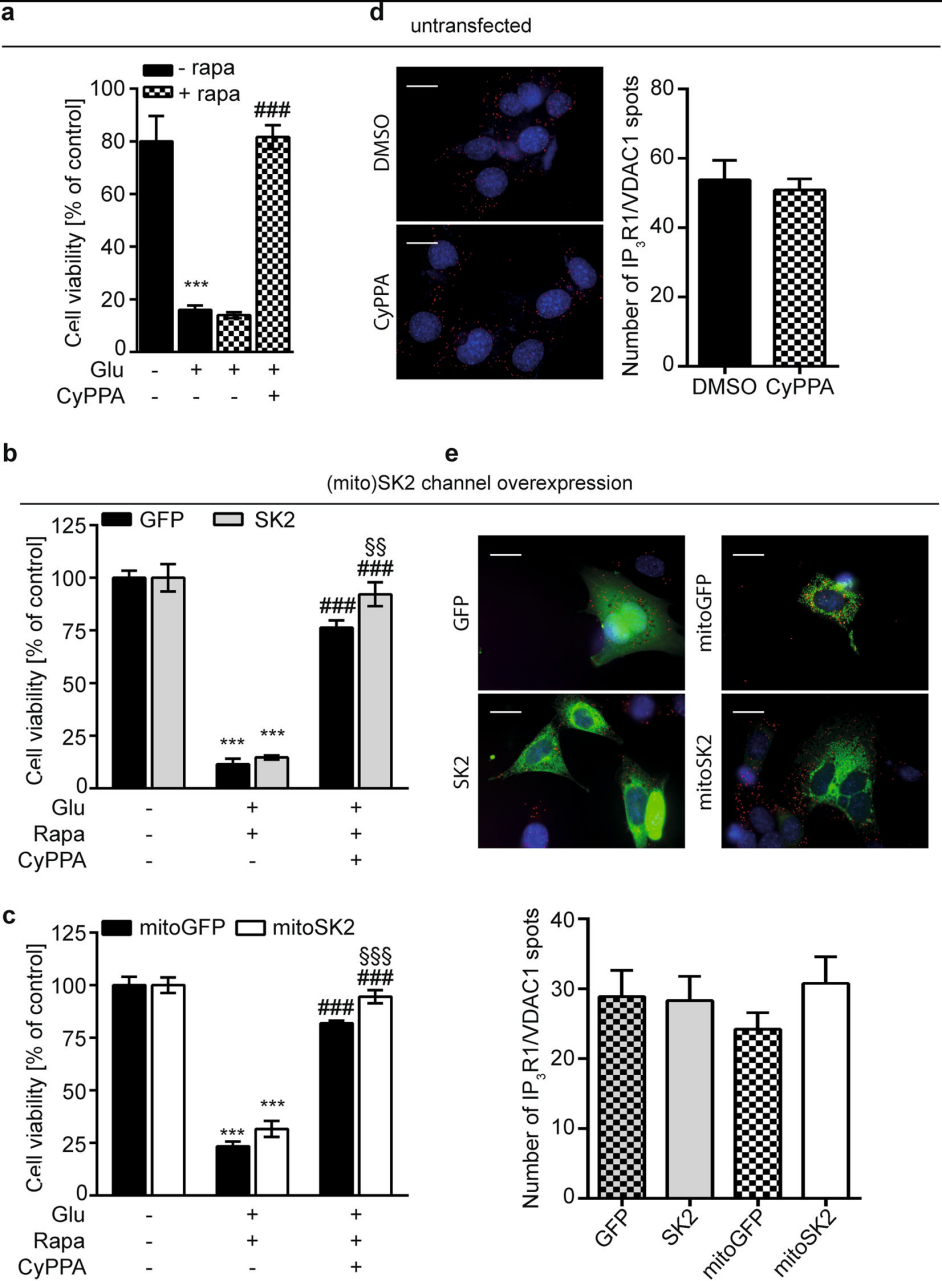
Activation of SK channels protects against neuronal toxicity in conditions of enhanced EMC without affecting physical ER–mitochondrial coupling. **a** MTT Assay in HT22 cells transfected with ER–mitochondrial linkers following treatment with glutamate (4 mM, 16 h) in the presence or absence of rapamycin (100 nM) and/or CyPPA (10 µM, gray bars). Data are presented as mean ± SD, *n* = 6, ***p < 0.0001. **b**, **c** MTT assay in HT22 cells transfected with linker plasmids (EML) and (**b**) GFP or SK2 or (**c**) mitoGFP and mitoSK2 plasmids following treatment with glutamate (4 mM, 16 h) in the presence or absence of rapamycin (100 nM) and/or CyPPA (10 µM). Data are presented as mean ± SD, *n* = 6–8, Student’s *t*-test, **p < 0.01, ***p < 0.0001, *compared to control, ^#^compared to glutamate+rapamycin, ^§^compared to GFP in the same treatment. In situ proximity ligation assay (PLA) in **d** HT22 cells treated for 24 h with vehicle (DMSO) or 10 µM CyPPA. In situ PLA in **e** HT22 cells transfected for 48 h with either GFP control plasmid or SK2 channel plasmid (left panels, light gray bars), or with mitoGFP control plasmid or mitoSK2 plasmid (right panels, white bars). Upper panels: representative images after in situ PLA, blue: DAPI, scale bar: 20 µM. Lower panels: quantification of IP_3_R1/VDAC1 spots on the analyzed pictures. Data are presented as mean ± SEM, *n* = 25–30 per condition

Here, we assessed the effects of SK channel activation in conditions of strengthened EMC on neuronal toxicity induced by glutamate. Therefore, we treated linker-transfected HT22 cells with rapamycin and challenged them with glutamate in the presence or absence of CyPPA. The treatment with CyPPA alone did not alter cell survival in cells transfected either with the Flipper control plasmid or the linkers, as assessed by cellular impedance measurements (Figure S3). In agreement with our previous study^17^, CyPPA protected against cell death induced by oxytosis. Here, we now show that neuroprotection was still present even in conditions of strengthened EMC (Fig. 4a). We also challenged linker-transfected HT22 cells that were co-transfected with either wild-type SK2 channels (SK2) or mitochondria-targeted SK2 channels (mitoSK2), with glutamate in the presence of rapamycin to induce linkage formation. Overexpression of either SK2 or mitoSK2 (Fig. 4b) combined with CyPPA preserved cell viability following oxytosis, and cell survival was enhanced in cells transfected with SK2/mitoSK2 compared to the GFP/mitoGFP control plasmids. These results indicate that the overexpression of SK2 channels increased CyPPA-mediated protection against glutamate toxicity. Further, we investigated if the enhanced neuroprotective action of SK2 channel activation and/or overexpression was based on effects on the physical EMC. Using a proximity ligation approach, we assessed IP_3_R1–VDAC1 interactions and found that neither CyPPA treatment (Fig. 4d) nor the overexpression of wild-type or mitochondrial SK2 channels (Fig. 4e) influenced IP_3_R1–VDAC1 interaction sites.

These findings show that the pharmacological activation of SK channels still provides protection even in conditions of enhanced EMC, indicating that CyPPA could overcome the additional damage through enhanced [Ca^2+^]_m_ and reduced mitochondrial respiration. Increasing the expression of both, wild-type and mitoSK2 channels provides an additive effect on CyPPA-mediated protection. Interestingly, neuroprotective SK2 channel actions were independent of the physical EMC.

## Discussion

Recent advances in the knowledge on the pathology of neurodegenerative diseases revealed a strong impact of EMC, MAM formation, and associated [Ca^2+^]_m_ transfer on mitochondrial metabolism and neuronal cell death^25–27^. Weakening the ER–mitochondrial interface has therefore emerged as a strategy to prevent the development of neurodegenerative diseases^28,29^. In the present study, we provide evidence that increasing EMC in neuronal HT22 cells potentiated cell death in a model of oxytosis. We further investigated the impact of SK channels on cell viability in these conditions, since pharmacological SK channel activation provided neuroprotection in different disease paradigms in vitro and in vivo by regulating Ca^2+^ homeostasis.

ER and mitochondria are physically linked mainly by a trimeric complex consisting of ER-bound IP_3_R, VDAC on the OMM and the molecular chaperone GRP75^11,30,31^. Recently, it has been shown that ER–mitochondrial contact points can be formed using drug-inducible fluorescent organelle linkers at the ER membrane and outer mitochondrial membrane, which bring ER and mitochondria into close proximity (<10 nM)^19^. In the current study, we expressed these drug-inducible EML in immortalized hippocampal HT22 cells, and confirmed the rearrangement of ER and mitochondria upon rapamycin treatment using live-cell imaging. Similar to Csordás and colleagues^19^, we observed full co-localization of the CFP-tagged ER plasmid and RFP-tagged mitochondrial plasmids, indicating a pronounced increase in EMC within minutes following addition of rapamycin.

ER–mitochondrial Ca^2+^ transfer and [Ca^2+^]_m_ load are critical factors to maintain mitochondrial function, with alterations in these processes promoting mitochondrial dysfunction. For instance, enhanced EMC provoked by the saturated fatty acid palmitate disrupted intracellular Ca^2+^ homeostasis, thereby promoting hepatotoxicity^32^, and enhanced VDAC-mediated [Ca^2+^]_m_ influx amplified cell death induction in HeLa cells^10,33^. Here, we utilized mitochondria-targeted GFP-aequorin^21^ measurements to confirm that strengthening EMC by inducing heterodimerization of the linkers in HEK293T cells specifically increased Ca^2+^ uptake into the mitochondria. Ca^2+^ signaling through the MAM interface also determines the cellular bioenergetic state, as shown for Ca^2+^ release through IP_3_R^20,34,35^ and the interaction of mitofusin 1 and mitofusin 2^36^. We observed that the increase in [Ca^2+^]_m_ influx, elicited by heterodimerization of the EML, was accompanied by a decrease in ATP production and in the mitochondrial reserve respiratory capacity. In contrast, in a model of ER stress, EMC was increased at early stages following ER stress induction, which was accompanied by an initial increase in mitochondrial respiration before it was attenuated concomitantly with the onset of cell death (20 h)^34^. The reason for these different effects may be that EMC induced by the bifunctional linkers was a rather fast and highly efficient process occurring within minutes compared to the ER stress-induced increase in EMC (1–4 h) observed by Bravo and colleagues^34^. Thus, we propose that basal mitochondrial respiration was stable without an initial increase due to the speed and strength of the artificial linkage induced by the linkers.

The intensity of ER–mitochondrial associations therefore also regulates local Ca^2+^ homeostasis, and stress-induced Ca^2+^ dysregulation in both organelles is detrimental^37,38^. To this end, we investigated cell survival following stress induction in HT22 cells expressing the bifunctional linkers to establish a stable platform for increased ER–mitochondrial interactions. Glutamate-induced cell death, starting approximately 12 h following induction, was more pronounced in cells transfected with the linkers compared to control cells. Thus, our findings indicate that EMC is a critical factor that potentiates neuronal damage generated by glutamate exposure.

SK channels, which are involved in controlling synaptic plasticity and neuronal firing, preserve cell survival in multiple disease paradigms by regulating Ca^2+^ signaling and mitochondrial function^39,40^. Despite their localization at the plasma membrane, recent studies have identified intracellularly expressed SK channels in the ER membrane and the IMM^16,17,39,41^ where they provide protection against ER stress and/or mitochondrial demise. Here, we show that activation of SK channels by the pharmacological SK2/3 channel activator CyPPA was able to protect against glutamate toxicity even in conditions of enhanced EMC that was associated with increased [Ca^2+^]_m_ uptake and impaired mitochondrial respiration. Interestingly, the overexpression of either wild-type or mitoSK2 channels enhanced the neuroprotective effects of CyPPA following glutamate treatment. Wild-type SK2 channels partially co-localized with the ER^16^ suggesting a contribution of SK channels from both, ER and mitochondria, to signaling along the MAM interface which might contribute to the observed protection during EMC enhancement. This concept is further supported by the fact that the physical association via IP_3_R1–VDAC1 interactions was not changed by SK channel activation and/or overexpression. These results highlight that SK channel activation is a promising approach to prevent neurodegeneration in conditions of glutamate toxicity even if neuronal cells are facing additional stress, such as increased [Ca^2+^]_m_ flux along the ER–mitochondrial axis and reduced mitochondrial respiration.

In conclusion, we show that strengthening EMC using genetically encoded linkers created a stable platform for ER–mitochondrial interactions, which enhanced [Ca^2+^]_m_ uptake and impaired mitochondrial bioenergetics, thereby potentiating neuronal cell death initiated by glutamate. SK channel activation by CyPPA, known to provide protection in different in vitro models of neurodegeneration, conferred protection against glutamate toxicity even when stress was pronounced due to an increase in EMC. These findings open a new platform to study SK channel-mediated protection in conditions of oxidative stress and mitochondrial dysfunction that are related to changes in ER–mitochondrial signaling.

## Materials and methods

### Cell culture

HT22 cells were cultured in Dulbecco’s modified Eagle Medium (Sigma Aldrich, Munich, Germany) supplemented with 10% heat-inactivated fetal calf serum (PAA Cölbe, Germany), 100 U/mL penicillin, 100 µg/mL streptomycin, and 2mM L-glutamine (Invitrogen, Karlsruhe, Germany) at 37 °C and 5% CO_2_. Plasmid transfection was performed using the attractene transfection reagent according to the manufacturer’s fast-forward protocol (Qiagen, Hilden, Germany). HT22 cells were transfected with 1.2 µg plasmid DNA and grown in a 6-well plate for 48 h followed by re-seeding into the appropriate plate format for subsequent experiments. HEK293T cells were transfected with 400 ng plasmid DNA and grown in a white-walled 96-well plate for 48 h. HT22 cells were challenged with glutamate (Sigma Aldrich, Munich, Germany).

### Cell viability

Cell viability was determined based on the metabolic activity using the MTT assay at a final concentration of 0.5 g/L by incubation for 1 h at 37 °C, followed by removal of the MTT and at least 1 h incubation at −20 °C. After dissolving the resulting formazan in DMSO, the absorbance of each well was determined with the Synergy H1 Multi-Mode reader (Biotek, LA, USA) at 570 nM and at 630 nM. Alternatively, cell viability was monitored in real-time with cell impedance measurements, using the xCELLigence system^42^ (ACEA Biosciences). Cell impedance was normalized to the time of treatment (normalized cell index), which is defined as the starting point (*t* = 0 h) of the experiment.

### Visualization of ER–mitochondrial contacts in living cells

To evaluate ER–mitochondrial contact formation upon transfection with TOM70-FKBP-mRFP and either ER-9xFRB-CFP (both linkers, depicted as EML) or ER-Flipper-GFP (depicted as FL) followed by the rapamycin-induced heterodimerization, widefield fluorescence microscopy (DeltaVision Elite) studies were performed. Transfected HT22 cells (2 × 10^5^ cells/well) were grown on Ø25 mm coverslips (Menzel-Gläser, Thermo Fisher, Landsmeer, The Netherlands). After image acquisition in untreated conditions, rapamycin (100 nM) was applied, and images were taken again after 5 min and 10 min at 60× magnification, pixel size 6.5 μm × 6.5 μm, 2560 × 2160 pixels, speed 400 fps at 512 × 512 pixel. The software adopted for acquisition and integrated deconvolution was softWoRx (on Linux CentOS 6.3 platform). Image overlays were done using the ImageJ software.

### [Ca^2+^]_m_ measurements

HEK293T cells were transfected with wild type and mutant mitochondrial GFP-aequorin (mtGA^wt^, mtGA^mut^) for 48 h in a clear bottom, white-walled 96-well plate (Greiner Bioscience, Frickenhausen, Germany). Briefly, mtGA constructs were reconstituted with native coelenterazine (Biotium, VWR Technologies, Darmstadt, Germany) in medium for 2 h. Cells were then washed with PBS followed by addition of 100 µL internal buffer (140 mM KCl, 1 mM KH_2_PO_4_, 1 mM MgCl_2_, 10 mM glucose, 0.1 mM EGTA, 20 mM HEPES, 8 mM Na-succinate, 4 mM Na-pyruvate, 4 mM MgATP). Ca^2+^ uptake was initiated by addition of 50 mM CaCl_2_ or 500 µM carbachol in internal buffer supplemented with 2.5 mM MgEDTA following background measurement for 5 s. Final sensor saturation was performed by addition of lysis buffer (140 mM KCl, 10 mM CaCl_2_, 1% TritonX100). Luminescence was recorded using the FluoStar OPTIMA plate reader (BMG Labtech, Offenbach, Germany). Each condition was measured at least in triplicate, and Ca^2+^ uptake was calculated as the total luminescent counts per second (L) from the time of stimulation (*t* = 5 s) until the time of final cell lysis (*t* = 30 s). Values were normalized to the initial value. For mtGA^wt^ measurements, these values were additionally normalized to the total luminescence (*t* = 0 s until *t* = 45 s). The sensor was calibrated with increasing CaCl_2_ concentrations (0, 10, 20, 30, 40, 50, 100 mM) resulting in a dose-dependent increase in the luminescent signal as determined by cps and the normalized luminescence (*L*_*t* = 5–30_/*L*_total_).

### Seahorse XF analysis

HT22 cells were transfected with mitochondrial linkers and either ER-Flipper control plasmid or the ER linker in Seahorse XF 96-well plates (Seahorse Biosystems, Agilent Technologies, Waldbronn, Germany). Prior to the measurement, the medium was removed and replaced by 180 μL assay medium containing 4.5 g/L glucose, 2 mM L-glutamine, 1 mM pyruvate (pH 7.35) for 1 h at 37 °C. The OCR was analyzed using the Seahorse XF Biosystem. Three baseline measurements (3 min mix, 0 min delay, 3 min measure = 3/0/3) were recorded followed by injection of rapamycin (50–200 nM) or medium and measurement for 2 h (3/5/3). Following EMC, mitochondrial function was assessed by injection of 4 µM oligomycin (3/0/3), 50 µM DNP (3/0/3), and 150 nM rotenone, 1 µM antimycin A, and 50 mM 2-DG (3/0/3). After injection of each compound, the OCR was determined and values were normalized before injection into port A.

### Quantitative real-time PCR

CHOP and GAPDH expression were analyzed using quantitative real-time PCR (qPCR). Therefore, total RNA was extracted from HT22 cells transfected with TOM70-mRFP and either ER-Flipper GFP (FL) or ER-CFP (EML) and treated with 100 nM and 200 nM rapamycin for 6 h, using Trizol RNA extraction (TRI Reagent Solution, Applied Biosystems, Landsmeer, Netherlands). The cDNA was synthesized from 500–1000 ng RNA using the Reverse Transcriptase System (Promega, Madison, WI, USA), and the following protocol: 10 min 25 °C, 45 min 42 °C, 5 min 99 °C. The qPCR was performed with SYBR Green (Roche Diagnostics, Almere, Netherlands) and the following protocol including a final step to generate the melting curve: 2 min 95 °C, 10 min 95 °C, 45× (30 s 95 °C, 30 s 60 °C, 30 s 72 °C), 30 s 95 °C, 30 s 55 °C, 30 s 95 °C. The qPCR was performed in an Eco Illumina (Illumina, Eindhoven, Netherlands). For analysis, the LinRegPCR software^43^ was used to calculate N0 values which were normalized to N0 of *Gapdh* as an internal control. Data are acquired in duplicates from three independent experiments.

The following murine primers were used: *Chop (*5′ CATACACCACCACACCTGAAAG 3′ and 3′ CCGTTTCCTAGTTCTTCCTTGC 5′) and *Gapdh (*5′ GGAGAGTGTTTCCTCGTCCC 3′ and 3′ ATGAAGGGGTCGTTGATGGC 5′).

### In situ proximity ligation assay

An optimized in situ proximity ligation assay (PLA) targeting the IP_3_R/VDAC1 complex at the MAM interface was performed, as previously described^44,45^. Briefly, following CyPPA treatment or transfection with GFP/SK2 constructs, HT22 cells cultured on 35 mm glass bottom dishes (MatTek, Ashland, MA, USA) were fixed with 4% PFA for 10 min, permeabilized using 0.3% PBS/TritonX-100 for 30 min, and saturated. The in situ PLA was performed according to the manufacturer’s protocol: VDAC1 (mouse anti-VDAC1 primary antibody) and IP_3_R1 (rabbit anti-IP_3_R1 primary antibody) were probed followed by addition of the secondary antibodies anti-mouse and anti-rabbit IgG (PLA probe MINUS and PLUS) conjugated to complementary oligonucleotide extensions. If target proteins were within 40 nM proximity, the oligonucleotides could hybridize with the connector oligonucleotides to form a circular DNA template, which was ligated and amplified to create a single-stranded DNA product. The DNA product is covalently attached to one of the proximity probes to allow detection of hybridized Texas red-labeled oligonucleotide probes. Each red fluorescent dot represents a site of IP_3_R1–VDAC1 interaction. Duolink II mounting medium containing DAPI 18 (Sigma Aldrich, Munich, Germany) was used for mounting, and preparations were analyzed with a Zeiss inversed fluorescent microscope at 63× magnification. Quantification of signals (number of red dots per cell) was done using the BlobFinder software. Experiments were performed in triplicate, *n* = 25–30 pictures per conditions.

### Statistical analysis

Statistical significance was assessed using the unpaired Student’s *t*-test or Analysis of variance (ANOVA) and Scheffé’s test for multiple comparisons, unless otherwise stated. *p*-values indicating statistically significant differences between the mean values are defined as follows: *p < 0.05, **p < 0.01, and ***p < 0.001.

## Electronic supplementary material

Supplementary figure Legends

Supplementary figure 1

Supplementary figure 2

Supplementary figure 3
