# Supplementary material for: SK channel activation is neuroprotective in conditions of enhanced ER–mitochondrial coupling
Source: Cell Death Dis. 2018 May 22;9(6):593. doi: 10.1038/s41419-018-0590-1 (PMC5964177; doi:10.1038/s41419-018-0590-1)
Supplement: Supplementary file 1 — Supplementary figure Legends [file 41419_2018_590_MOESM1_ESM.docx]

## Supplementary figure 1. [Ca^2+^]_m_ measurements in HEK293T cells.

(a) Representative measurement of [Ca^2+^]_m_ uptake in HEK293T cells transfected with mtGA^wt^ stimulated with 0-100mM CaCl_2_. Data are presented as mean±SD, n=3-4. (b) Calibration of the mitochondrial calcium sensor by increasing CaCl_2_ concentrations. Data are represented as mean±SD, n=3-4, normalized to total luminescence (L_t=5-30_/L_total_). (c) MTT assay in ER (grey bars) or linker-transfected (blue bars) HEK293T cells treated with the indicated rapamycin concentrations. Data are presented as mean±SD, n=6-8.

## Supplementary figure 2. Effect of enhanced EMC on cell viability.

(a) xCELLigence measurement of HT22 cells transfected with Flipper control plasmid (FL) or linkers (EML) following treatment with rapamycin (50, 100, 150, 200nM). Data are presented as mean±SD, n=6. (b) Relative mRNA expression of *CHOP* in HT22 cells transfected with TOM70-FKBP12-mRFP and Flipper control plasmid (named FL, grey bars) or together with CFP-FRB-ER (named EML, blue bars) following the application of 100nM and 200nM rapamycin. Data are normalized to GAPDH mRNA expression and shown as mean±SD, n=3 per condition.

## Supplementary figure 3. Effect of CyPPA on cell proliferation.

xCELLigence measurement of HT22 cells transfected with Flipper control plasmid (FL) or linkers (EML) following treatment with CyPPA (10, 25, 50µM). Data are presented as mean±SD, n=6.
