# Supplementary figures and images for: SK channel activation is neuroprotective in conditions of enhanced ER–mitochondrial coupling
Source: Cell Death Dis. 2018 May 22;9(6):593. doi: 10.1038/s41419-018-0590-1 (PMC5964177; doi:10.1038/s41419-018-0590-1)

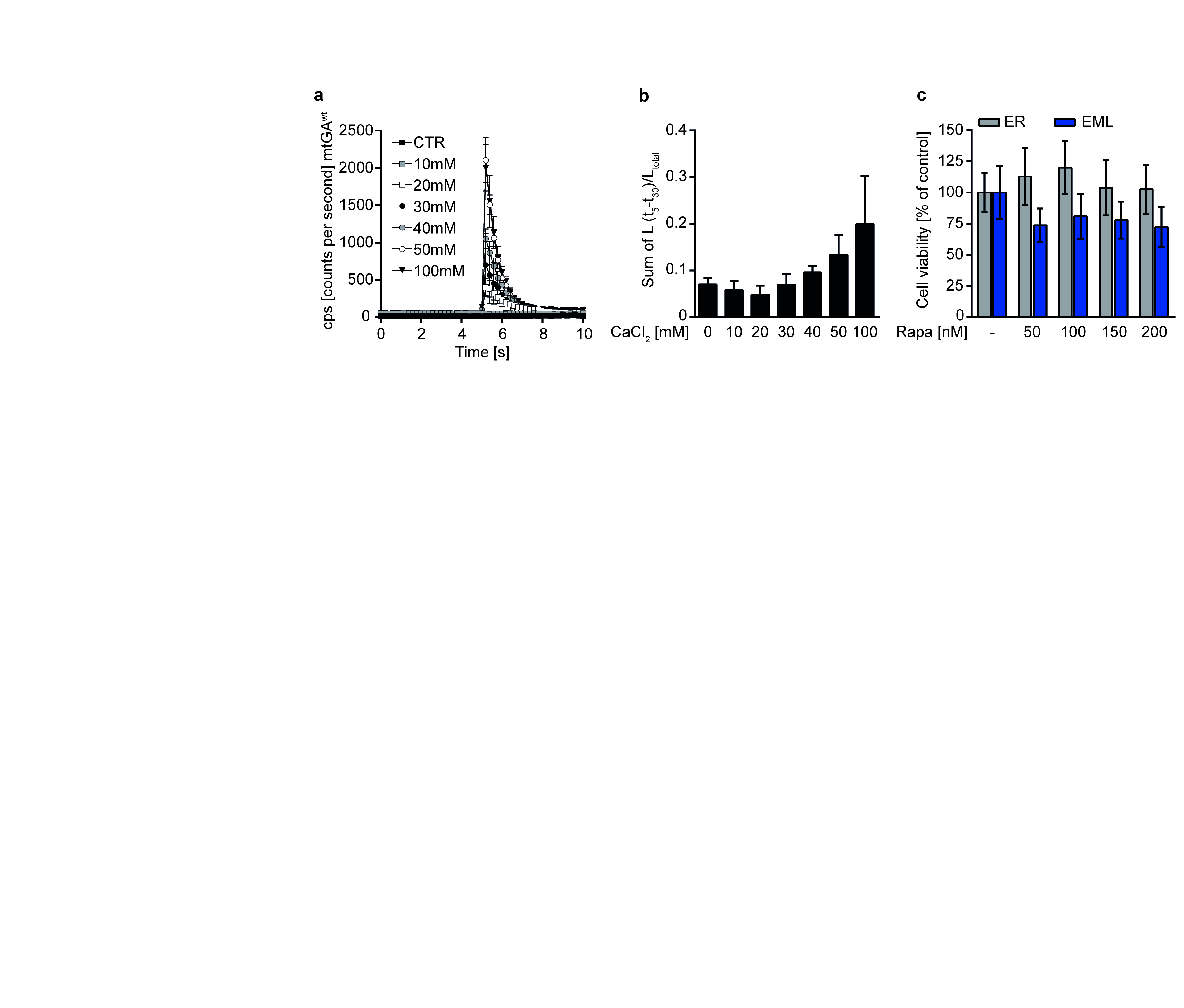

Supplement: Supplementary file 2 — Supplementary figure 1 [file 41419_2018_590_MOESM2_ESM.tif]

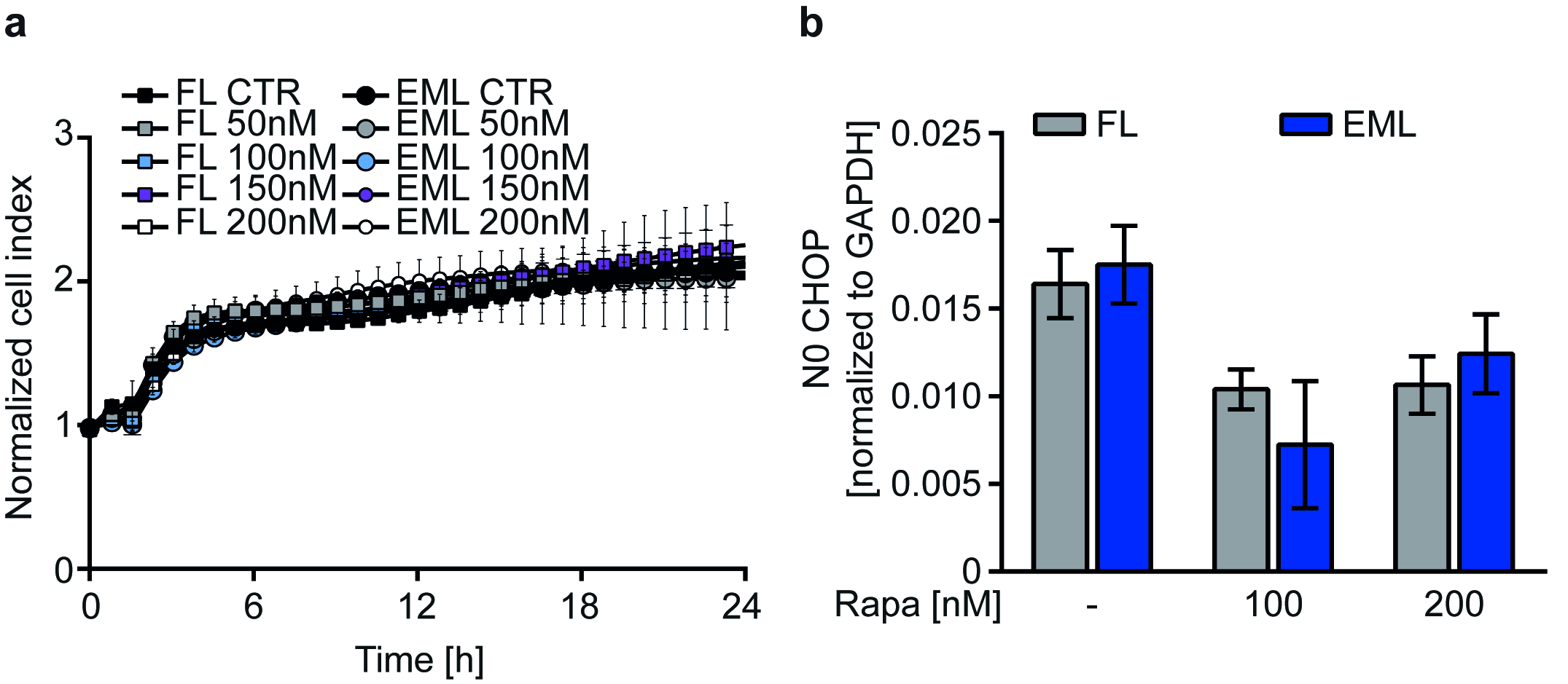

Supplement: Supplementary file 3 — Supplementary figure 2 [file 41419_2018_590_MOESM3_ESM.tif]

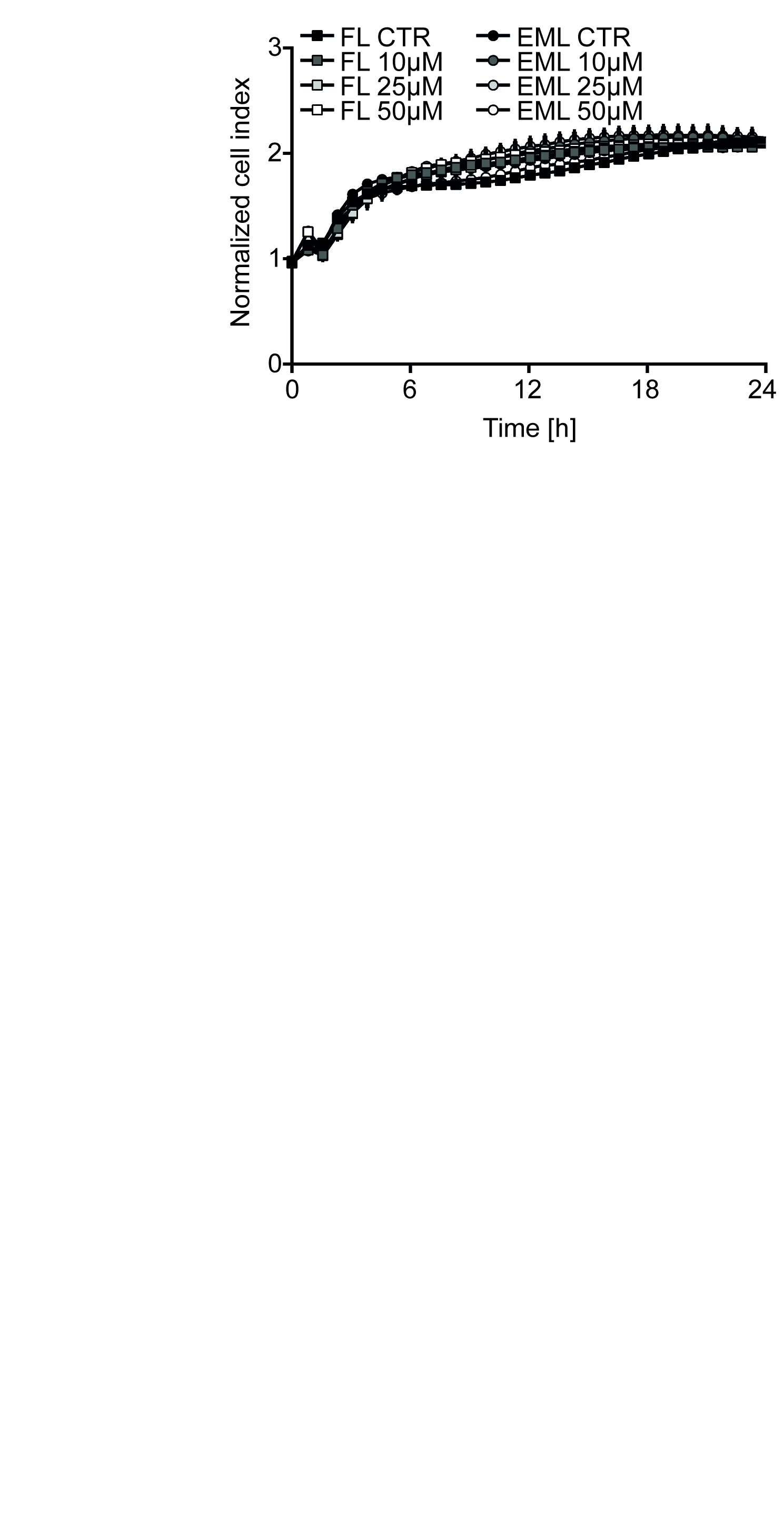

Supplement: Supplementary file 4 — Supplementary figure 3 [file 41419_2018_590_MOESM4_ESM.tif]
